# Magnitude of fetal macrosomia and its associated factors at public health institutions of Hawassa city, southern Ethiopia

**DOI:** 10.1186/s13104-018-4005-2

**Published:** 2018-12-13

**Authors:** Andargachew Kassa Biratu, Negash Wakgari, Birhanu Jikamo

**Affiliations:** 1 Department of Midwifery, College of Medicine and Health Sciences, Hawassa University, Hawassa, Ethiopia; 2 School of Public Health, College of Medicine and Health Sciences, Hawassa University, Hawassa, Ethiopia

**Keywords:** Fetal macrosomia, Magnitude, Hawassa

## Abstract

**Objectives:**

This study aimed to determine the magnitude of fetal macrosomia and associated factors at public health institutions of Hawassa city, southern Ethiopia.

**Results:**

In this study, the magnitude of fetal macrosomia found to be 11.86%. Being a male (AOR = 2.2, 95% CI 1.1–4.2), ≥ 37 weeks gestational age (AOR = 6.0, 95% CI 3.1–11.1) and having previous history of fetal macrosomia (AOR = 14.5, 95% CI 7.2–29.2) had a higher odds of fetal macrosomia. Moreover, the magnitude of fetal macrosomia is found be in the global range. Sex of the child, previous history of fetal macrosomia and gestational age were significantly associated with fetal macrosomia. The obstetric care providers should assess all pregnant women for history of fetal macrosomia which would help them to be prepared for the managements of maternal and perinatal complications.

**Electronic supplementary material:**

The online version of this article (10.1186/s13104-018-4005-2) contains supplementary material, which is available to authorized users.

## Introduction

Macrosomia, which is defined as a birth weight > 4000 g [1], is negatively affect both maternal and neonatal outcome [2, 3]. The prevalence of fetal macrosomia varied from region to region [4–13], due to variety of their contributory factors investigated in different studies [6, 7, 9–15]. Globally, macrosomia affects 3 to 15% of all pregnancies [16]. In developed world the magnitude of macrosomia is ranging from 5 to 20% of all births [17]. Fetal macrosomia complicates delivery process for both mothers and neonates [18]. Macrosomic baby have a higher threat of developing both short and long term health outcomes; including birth asphyxia, still birth, obesity and metabolic disorders [19–21]. Infant macrosomia also associated with higher risk of certain cancers [22–25]. In addition, shoulder dystocia, skeletal injuries, meconium aspiration, hypoglycemia, and fetal death are reported to be associated with fetal macrosomia [16, 26]. Similarly, a long term health effects contributed by macrosomia includes type 2 diabetes mellitus, hypertension, and obesity in adulthood [27]. Moreover, postpartum hemorrhage, perineal tears, prolonged labor, shoulder dystocia, uterine rupture and maternal deaths are the major maternal complications contributed by fetal macrosomia [7, 21]. Furthermore, labor augmentation with oxytocin, cesarean delivery, infection, failed instrumental deliveries, wound complications, thromboembolic events, and anesthetic accidents are other maternal complications of macrosomia [28–30].

Compared with developed countries, researches on macrosomia in developing world such as Ethiopia, particularly in the study settings are insufficient. Also, adverse maternal and neonatal outcomes due to macrosomia can lead to additional maternal and neonatal risks in a resource-limited country because of the restricted availability of basic emergency obstetric and newborn care [30]. Understanding certain modifiable risk factors for macrosomia such as high pre-pregnancy body mass index, high blood glucose level during pregnancy and low level of pre-gestational physical activity are crucial for health care providers to prevent macrosomia by suitable diet and insulin therapy [8, 10, 11, 13, 31]. Hence, this study aimed to determine the magnitude of fetal macrosomia and associated factors at public health institutions of Hawassa city, southern Ethiopia.

## Main text

### Study design, setting and population

A cross-sectional study was conducted among women gave birth in public health institution of Hawassa city administration. Hawassa city administration is a capital city of southern Nation, Nationality and People regional state located 275 km south from Addis Ababa, capital city of Ethiopia. The city has been structured by 7 urban sub-cities collectively having 21 kebeles and 1 rural sub-city with 11 kebeles. Regarding health institution, the city has 83 health institutions (32 public and 51 private clinics). There are about a total of 852 health professionals working in the randomly selected primary health institution of the city. Together with about 600 health professionals working in Hawassa University comprehensive specialized hospital, the number reached 1452. The source populations were all pregnant women living in the Hawassa city administration. Whereas, the study population were those laboring mother’s who attended the randomly selected public health institutions for delivery service during the study period. Mothers with a twin pregnancy were excluded from the study.

### Sample size and sampling procedure

The sample size was calculated using single population proportion formula: The proportion (p) = 50% (no published works in the study setting), 95% confidence level = 1.96, 5% of absolute precision. With a non response rate of 10% and a design effect of two for multistage sampling, the total sample size became 580.

Accordingly, multistage sampling technique was employed to include 580 study participants. At first stage, out of the 11 public health institutions, five of them were selected using simple random sampling technique. The selected health institution include: Hawassa University comprehensive specialized hospital, Adare General Hospital, Adare primary health center, Millennium primary health center, and Title primary health center. Then after, the numbers of deliveries’ conducted 6 months before the study period in the selected health facilities were assessed to be used as a base to allocate the sample to the selected health facility. So that, the number of deliveries reported from Hawassa University comprehensive specialized hospital was 4780, Adare General Hospital 2022, Adare primary health center 313, Millennium primary health center 311, and Title primary health center 61. Hawassa University comprehensive specialized hospital and Adare General Hospital are the only public health hospitals providing comprehensive essential obstetric care in the city. At the second stage, we applied a systematic random sampling method to identify and include all the study participants. The samples were taken proportionate to the number of expected deliveries from each selected public health institution.

### Data collection tools and procedure

Interviewer-administered questionnaire were used to collect data. Relevant literature was reviewed to develop the tool and to include all the possible variables that address the objective of the study [4–8, 10–15, 29–31]. The instrument was first developed in English and translated into Amharic and then back to English to check the accuracy. Before the actual data collection, the questionnaire was tested on 10% of health care providers working in public health institutions of a nearby town called Shashemene.

Every woman who came to the selected public health institution for delivery purpose during the data collection period was interviewed. In addition to the interview, the data collectors abstracted clinical data by reviewing the mother and the babies’ medical records. They also measured and confirmed the baby’s birth weight, and conducted selected examinations for which they get additional training. The data was collected by 5 B.Sc. female midwives and 5 B.Sc. female nurses after 1-day training about informed consent, techniques of interviewing, data collection procedures, and different sections of the questionnaire. Two health officers were assigned as supervisors for the data collectors. Overall supervision also made by the principal investigator.

### Data processing and analysis

All collected questionnaires were rechecked for completeness and coded. Then these data were entered and cleaned using SPSS version 20 for analysis. Bivariable and multivariable logistic regression was employed to identify an association between the independent predictors and the outcome variable. Those factors found with their P-value ≤ 0.20 in the bi-variable logistic regression model were fitted into the multivariable logistic regression model to control the effect of confounding variables. Multivariable analysis using standard logistic regression technique was done to evaluate the independent effect of each covariate by controlling the effect of others. Variable having P-value of less than 0.05 in the multivariable logistic regression analysis was considered as significantly associated factors of fetal macrosomia. The adjusted odds ratios with the 95% Confidence Intervals were reported. Before the actual logistic regression analysis was done, the necessary assumption of logistic regression model was checked by using Hosmer–Lemeshow test of goodness of fit.

### Results

#### Socio-demographic characteristics of the respondents

A total of 580 respondents were included in the study (response rate = 100%). Most of them 560 (96.6%) were married. The majority, 492 (84.6%) were between the age of 20–34 years and more than half, 325 (56%) had primary education. More than two-thirds 456 (78.6%) of the respondents had less than five family size (Additional file 1: Table S1).

#### Fetal macrosomia and risk factors

In this study, 63 (11.86%) of delivered fetus were macrosomic and 67 (11.55%) of them were low birth weight (< 2.5 kg). About two-thirds, 450 (77.6%) of the newborn baby had a normal birth weight (2.5–3.9 kg). Majority, 465 (80.2%) of the women had preconception Body Mass Index (BMI) of 18.5–24.9. More than half 330 (56.9%) of child’s sex were male. Most 519 (89.5%) of the gestational age of the pregnancy were term. Similarly, 540 (93.2%) of the respondents were not diagnosed with any chronic disease. Moreover, 523 (90.2%) of the women had no history of macrosomic infants (Table 1).

**Table 1:** Frequency distribution of selected risk factors for macrosomia in Hawassa city, South Ethiopia, 2017

Risk factors to macrosomia	Category	Frequency (n)	Percent (%)
BMI	18.5–24.9	465	80.2
	< 18.5	76	13.1
	> 24.9	39	6.7
Age of the mother (Advanced maternal age) (years)	< 20	45	8
	20–34	492	84.6
	35–49	43	7.4
Sex of the child (male child)	Male	330	56.9
	Female	250	43.1
Gestational (post term pregnancy)	Preterm	58	10.0
	Term	519	89.5
	Post term	3	0.5
Diagnosed with chronic diseases (diabetes)	No chronic disease	540	93.2
	Diagnosed with chronic diseases other than diabetes	37	6.3
	Diagnosed with diabetes	3	0.5
Previous history of macrosomia (previous macrocosmic infant)	No history of previous macrocosmic infant	523	90.2
	Had history of infant with macrosomia	57	9.8
Weight gain during current pregnancy (excessive weight gain) (kg)	≤ 13	509	87.8
	> 13	71	12.2
Para (multi parity)	I	306	52.8
	II–IV	236	40.7
	≥ V	38	6.6

No data of maternal birth weight

#### Adverse maternal and infant outcome in relation to fetal macrosomia

Most, 57 (10%) of macrosomic infants had normal and alive pregnancy outcome in relation to child. Among women who had normal pregnancy and child birth, 60 (10%) of them were macrosomic. Similarly, 46 (8%) of macrosomic infants had delivered by spontaneous vaginal delivery (Table 2).

**Table 2:** The status of adverse maternal and infant related birth outcomes in relation to delivery of macrosomic infant, Hawassa, South Ethiopia 2017

Birth outcomes	Macrosomia		Total
	No	Yes	
Outcome of pregnancy related to the child			
Normal and alive	482 (83%)	57 (10%)	539 (93%)
Stillbirths	13 (2%)	1 (0%)	14 (2%)
Preterm births	17 (3%)	0 (0%)	17 (3%)
Intrauterine growth retardation	3 (1%)	0 (0%)	3 (1%)
Neonatal death	2 (0%)	0 (0%)	2 (0%
Congenital anomaly	1 (0%)	4 (1%)	5 (1%)
Outcome of pregnancy related to the mother			
Normal pregnancy and child birth	460 (79%)	60 (10%)	520 (90%)
Anemia	11 (2%)	0 (0%)	11 (2%)
Pregnancy induced hypertension	24 (4%)	1 (0%)	25 (4%)
Gestational diabetes mellitus	2 (0%)	1 (0%)	3 (1%)
Antepartum hemorrhage	7 (1%)	0 (0%)	7 (1%)
Postpartum hemorrhage	9 (2%)	0 (0%)	9 (2%)
Others^a^	4 (1%)	1 (0%)	5 (1%)
Mode of delivery			
Spontaneous vaginal delivery	415 (72%)	46 (8%)	461 (79%)
Instrumental delivery	10 (2%)	1 (0%)	11 (2%)
Caesarean section	92 (16%)	16 (3%)	108 (19%)

^a^Perineal tears, prolonged labor

#### Factors associated with fetal macrosomia

In the bivariate analysis the factors found to be significantly associated with fetal macrosomia were: sex of the child, gestational age, previous history of infant with macrosomia and monthly income. However, in the multiple logistic regression analysis: sex of the child, gestational age and previous history of infant with macrosomia were significantly associated with the magnitude of fetal macrosomia.

Being a male was about 2 times more likely to be associated with fetal macrosomia than being a female (AOR = 2.2, 95% CI 1.1–4.2). Similarly, being a gestational age of 37 weeks and above were about 6 times more likely of fetal macrosomia (AOR = 6.0, 95% CI 3.1–11.1). Moreover, having previous history of fetal macrosomia also significantly associated with fetal macrosomia (AOR = 14.5, 95% CI 7.2–29.2) (Table 3).

**Table 3:** Factors associated with fetal macrosomia among deliveries attended in public health institutions of Hawassa, South Ethiopia 2017

Risk factors to macrosomia	Birth weight		Odds ratios (OR)	
	Macrosomia	No macrosomia	COR (95% CI)	AOR (95% CI)
Sex of the child				
Male	47 (8.1%)	283 (48.8%)	2.4 (1.3–4.4)	2.2 (1.1–4.2)*
Female	16 (2.8%)	234 (40.4%)	1	1
Gestation (weeks)				
≥ 37	37 (6.4%)	96 (16.6%)	6.2 (3.6–10.8)	6.0 (3.1–11.1)*
< 37	26 (4.8%)	421 (72.6%)	1	1
Previous history of infant with macrosomia				
Had infant with macrosomia	30 (5.1%)	27 (4.7%)	16.5 (8.8–30.9)	14.5 (7.2–29.2)*
No history of previous infant with macrosomia	33 (5.7%)	490 (84.5%)	1	1
Monthly income in Ethiopian Birr (ETB)				
< 1000	38 (6.6%)	136 (23.5%)	0.43 (0.21–0.88)	0.49 (0.21–1.12)
1001–2000	25 (4.3%)	94 (16.2%)	0.71 (0.35–1.44)	0.59 (0.24–1.41)
2001–3675	34 (5.9%)	108 (18.6%)	0.52 (0.26–1.10)	0.53 (0.22–1.35)
> 3675	38 (6.6%)	107 (23.5%)	1	1

* P < 0.05

### Discussion

This study attempted to determine the magnitude of fetal macrosomia and associated factors at public health institutions of Hawassa city, southern Ethiopia. The magnitude of fetal macrosomia found to be 11.86% (95% CI 7.8, 15.2). This finding is consistent with the studies conducted in Algeria (10.9%) [4], Turkey (8.6%) [10], Iran (11.8%) [11], Tunisia (8.1%) [12] and Nigeria (8.0%) [32]. Moreover, the present study finding is lower than the studies done in Lithuania (24.4%) [6] and Mexico (18.6%) [13]; while, it is higher than the studies conducted in Tanzania (2.3%) [7], 14 provinces in China (7.3%) [8], South–South Nigeria (7.4%) [21], Tigray, Northern Ethiopia (6.68%) [9] and Hong Kong, China (2.89%) [29]. This variation might be due to difference in the socio-demographic characteristics of the respondents, study design, data collection procedure and type of study participants. For instances, in the study of Lithuania all respondents participated in the study were women with gestational diabetes, which could increase the magnitude of macrosomia [7, 18].

In the current study, there is a significant association between sex of the child and macrosomia. Those children who were male had 2.2 times greater odds of macrosomia than those who were female. This is similar with studies conducted in Turkey [10] and China [8, 15]. Similarly, being a gestational age of 37 weeks and above was also significantly associated with fetal macrosomia. This is consistent with study done in Norway [32] and Lithuania [6]. Moreover, previous history of macrosomia was significantly associated with fetal macrosomia. This finding is in line with the studies conducted in Iran [11, 18] and Lithuania [6].

In this study, the magnitude of fetal macrosomia is found be in the global range. Sex of the child, previous history of fetal macrosomia and gestational age were significantly associated with fetal macrosomia. The obstetric care providers should assess all pregnant women for history of fetal macrosomia which would help them to be prepared for the managements of maternal and perinatal complications.

## Limitations

This study has some limitations: Firstly, the study design did not actually investigate for modifiable risk factors such as high pre-pregnancy body mass index, high blood glucose level during pregnancy and low level of pregestational physical activity. Hence, the results of the study had a limited clinical value. Secondly, qualitative aspects of data were not included in this study to explore some determinant factors and to triangulate the finding of the quantitative data. Lastly, as the study is cross-sectional in design, it may not create true causal relationship between fetal macrosomia and its associated factors.

## Additional file

**Additional file 1: Table S1.** Socio-demographic characteristics of women who gave birth in public health institutions of Hawassa (n = 580), South Ethiopia 2017.

## Abbreviation

BMI: body mass index

## References

[1] World Health Organization Sustainable Development and Healthy Environments. International statistics classification of diseases and related health problems, Geneva, Switzerland. 1999. http://www.who.int/occupational_health/. Accessed 26 March 2018.

[2] Alberman E (1991). Are our babies becoming bigger?. J R Soc Med.

[3] Blondel B, Kermarrec M (2011). La situation perinatale en France en 2010. Etude et resultats..

[4] Mai AH, Abbassia D (2014). The prevalence of fetal macrosomia at the specialized hospital of gynecology and obstetrics of Sidi Bel Abbes (West Of Algeria). J Nutr Food Sci..

[5] Shan X, Chen F, Wang W, Zhao J, Teng Y, Wu M (2014). Secular trends of low birthweight and macrosomia and related maternal factors in Beijing, China: a longitudinal trend analysis. BMC Pregnancy Childbirth..

[6] Bukelskiene Z, Naskauskiene G, Visockiene Z (2016). Risk factors for fetal macrosomia in gestational diabetes. Endocr Abstr.

[7] Said AS, Manji KP (2016). Risk factors and outcomes of fetal macrosomia in a tertiary centre in Tanzania: a case-control study. BMC Pregnancy Childbirth.

[8] Li G, Kong L, Li Z, Zhang L, Fan L, Zou L (2014). Prevalence of macrosomia and its risk factors in china: a multicentre survey based on birth data involving 101,723 singleton term infants. Paediatr Perinat Epidemiol.

[9] Mengesha HG, Wuneh AD, Weldearegawi B, Divya L, Selvakumar (2017). Low birth weight and macrosomia in Tigray, Northern Ethiopia: who are the mothers at risk?. BMC Pediatr.

[10] Usta A, Usta CS, Yildiz A, Ozcaglayan R, Dalkiran ES, Savkli A (2017). Frequency of fetal macrosomia and the associated risk factors in pregnancies without gestational diabetes mellitus. Pan Afr Med J.

[11] Mahnaz M, Khalkhalirad A, Rossta S, Rezapour P (2014). Evaluation of the prevalence of macrosomia and the maternal risk factors. Iran J Neonatol..

[12] Mallouli M, Derbel M, Ingrid A, Sahli J, Zedini C, Ajmi T (2017). Associated outcomes to fetal macrosomia: effect of maternal diabetes. Tunis Med.

[13] García-De la Torre JI, Rodríguez-Valdez A, Delgado-Rosas A (2016). Risk factors for fetal macrosomia in patients without gestational diabetes mellitus. Ginecol Obstet Mex..

[14] Yu DM, Zhai FY, Zhao LY, Liu AD, Yu WT, Jia FM (2008). Incidence of fetal macrosomia and influencing factors in China in 2006. Chin J Prevent Med..

[15] Gu S, An X, Fang L, Zhang X, Zhang C, Wang J (2012). Risk factors and long-term health consequences of macrosomia: a prospective study in Jiangsu Province, China. J Biomed Res..

[16] Asplund CA, Seehusen DA, Callahan TL, Olsen C (2008). Percentage change in antenatal body mass index as a predictor of neonatal macrosomia. Ann Fam Med..

[17] Henriksen T (2008). The macrosomic fetus: a challenge in current obstetrics. Acta Obstet Gynecol Scand.

[18] Mohammadbeigi A, Farhadifar F, Soufi zadeh N, Mohammadsalehi N, Rezaiee M, Aghaei M (2013). Fetal macrosomia: risk factors, maternal, and perinatal outcome. Ann Med Health Sci Res..

[19] Boney CM, Verma A, Tucker R, Vohr BR (2005). Metabolic syndrome in childhood: association with birth weight, maternal obesity, and gestational diabetes mellitus. Pediatrics.

[20] Dyer JS, Rosenfeld CR, Rice J, Rice M, Hardin DS (2007). Insulin resistance in Hispanic large-for-gestational-age neonates at birth. J Clin Endocrinol Metab.

[21] Onyearugha CN, Ugboma H (2014). Macrosomia Prevalence and predisposing factors as seen at a university teaching hospital, South-South Nigeria. J Med Investig Pract..

[22] Sprehe MR, Barahmani N, Cao Y, Wang T, Forman MR, Bondy M (2010). Comparison of birthweight corrected for gestational age and birth weight alone in prediction of development of childhood leukemia and central nervous system tumors. Pediatr Blood Cancer.

[23] Harder T, Plagemann A, Harder A (2010). Birthweight and risk of neuroblastoma: a meta-analysis. Int J Epidemiol.

[24] Ognjanovic S, Carozza SE, Chow EJ, Fox EE, Horel S, McLaughlin CC (2010). Birth characteristics and the risk of childhood rhabdomyosarcoma based on histological subtype. Br J Cancer.

[25] Ahlgren M, Wohlfahrt J, Olsen LW, Sørensen TI, Melbye M (2007). Birthweight and risk of cancer. Cancer.

[26] Ahmed SR, Ellah MA, Mohamed OA, Eid HM (2009). Prepregnancy obesity and pregnancy outcome. Int J Health Sci (Qassim)..

[27] Hermann GM, Dallas LM, Haskell SE, Roghair RD (2010). Neonatal macrosomia is an independent risk factor for adult metabolic syndrome. Neonatology..

[28] Abolfazl M, Hamidreza TS, Narges MY (2008). Gestational diabetes and its association with unpleasant outcomes of pregnancy. Pak J Med Sci..

[29] Wong PY, Wk To W (2018). Risk factors and pregnancy outcomes of macrosomia: a retrospective cohort study. Hong Kong J Gynaecol Obstet Midwifery..

[30] Fuchs F, Bouyer J, Rozenberg P, Senat MV (2013). Adverse maternal outcomes associated with fetal macrosomia: what are the risk factors beyond birth weight?. BMC Pregnancy Childbirth.

[31] Voldner N, Frøslie KF, Bo K, Haakstad L, Hoff C, Godang K (2008). Modifiable determinants of fetal macrosomia: role of lifestyle-related factors. Acta Obstet Gynecol Scand.

[32] Kayode-Adedeji B, Egharevba O, Omoregbee H (2018). Prevalence of fetal macrosomia and neonatal complications in a Nigerian suburban hospital: a 5 year study. J Pediatr Neonatal Individ Med.

