# Supplementary material for: Magnitude of fetal macrosomia and its associated factors at public health institutions of Hawassa city, southern Ethiopia
Source: BMC Res Notes. 2018 Dec 13;11:888. doi: 10.1186/s13104-018-4005-2 (PMC6293502; doi:10.1186/s13104-018-4005-2)
Supplement: Supplementary file 1 — Additional file 1: Table S1. Socio-demographic characteristics of women who gave birth in public health institutions of Hawassa (n = 580), South Ethiopia 2017. [file 13104_2018_4005_MOESM1_ESM.docx]

Table S1፡ Socio-demographic characteristics of women who gave birth in public health institutions of Hawassa (n=580), South Ethiopia 2017

| **Variables** | **categories** | **Frequency(n)** | **Percent (%)** |
| --- | --- | --- | --- |
| **Age of the mother** | < 20 years | 45 | 8 |
|  | 20-34 years | 492 | 84.6 |
|  | 35-49 years | 43 | 7.4 |
| **Marital status** | Married | 560 | 96.6 |
|  | Single | 20 | 3.4 |
| **Education Status** | No formal education | 110 | 19 |
|  | Primary education (1-8 grade) | 325 | 56 |
|  | Secondary education (9-12 grade) | 97 | 16.7 |
|  | Tertiary (college or university) | 48 | 8.3 |
| **Occupation** | Housewife | 399 | 68.8 |
|  | Private business | 87 | 15 |
|  | Daily worker | 14 | 2.4 |
|  | Salaried employed | 61 | 10.5 |
|  | Student | 19 | 3.3 |
| **Monthly income** | <1,000 ETB | 174 | 30 |
|  | 1,001-2000 ETB | 119 | 20 |
|  | 2001- 3675 ETB | 142 | 25 |
|  | > 3675 ETB | 145 | 25 |
| **Total family size** | < 5 | 456 | 78.6 |
|  | 6 | 89 | 15.4 |
|  | >6 | 35 | 6 |
| **Residence** | Urban | 375 | 64.7 |
|  | Rural | 150 | 25.9 |
|  | Peri-urban | 55 | 9.4 |
| **Ethnicity** | Sidama | 232 | 40.0 |
|  | Oromo | 115 | 19.8 |
|  | Wolayeta | 106 | 18.3 |
|  | Gurage | 46 | 7.9 |
|  | Amhara | 42 | 7.2 |
|  | Other* | 39 | 6.7 |

*=Kembata, Hadiya, Gedio, Siltie
